# Olfactory Bulb Proteomics Reveals Widespread Proteostatic Disturbances in Mixed Dementia and Guides for Potential Serum Biomarkers to Discriminate Alzheimer Disease and Mixed Dementia Phenotypes

**DOI:** 10.3390/jpm11060503

**Published:** 2021-06-03

**Authors:** Mercedes Lachén-Montes, Ignacio Íñigo-Marco, Paz Cartas-Cejudo, Joaquín Fernández-Irigoyen, Enrique Santamaría

**Affiliations:** Clinical Neuroproteomics Unit, Navarrabiomed, Complejo Hospitalario de Navarra (CHN), Universidad Pública de Navarra (UPNA), Navarra Institute for Health Research (IdiSNA), Irunlarrea, 3, 31008 Pamplona, Spain; mercedes.lachen.montes@navarra.es (M.L.-M.); ignacio.inigo.marco@navarra.es (I.Í.-M.); pazcarce@hotmail.com (P.C.-C.); jfernani@navarra.es (J.F.-I.)

**Keywords:** mixed dementia, Alzheimer’s disease, vascular dementia, olfactory bulb, proteomics

## Abstract

The most common form of mixed dementia (MixD) is constituted by abnormal protein deposits associated with Alzheimer’s disease (AD) that coexist with vascular disease. Although olfactory dysfunction is considered a clinical sign of AD-related dementias, little is known about the impact of this sensorial impairment in MixD at the molecular level. To address this gap in knowledge, we assessed olfactory bulb (OB) proteome-wide expression in MixD subjects (*n* = 6) respect to neurologically intact controls (*n* = 7). Around 9% of the quantified proteins were differentially expressed, pinpointing aberrant proteostasis involved in synaptic transmission, nucleoside monophosphate and carbohydrate metabolism, and neuron projection regeneration. In addition, network-driven proteomics revealed a modulation in cell-survival related pathways such as ERK, AKT, and the PDK1-PKC axis. Part of the differential OB protein set was not specific of MixD, also being deregulated across different tauopathies, synucleinopathies, and tardopathies. However, the comparative functional analysis of OB proteome data between MixD and pure AD pathologies deciphered commonalities and differences between both related phenotypes. Finally, olfactory proteomics allowed to propose serum Prolow-density lipoprotein receptor-related protein 1 (LRP1) as a candidate marker to differentiate AD from MixD phenotypes.

## 1. Introduction

Alzheimer’s disease (AD) and vascular dementia (VaD) are the most common causes of dementia in the elderly [1]. In medical practice, the term mixed dementia (MixD) is mostly referred to cases where there are clinicopathological evidences of both AD and vascular disease [2]. Around 25% of demented patients have pure AD pathology, while more than 50% present different vascular lesions (such as micro/macroinfarcts, microhemorrhages, lacunar strokes, among others), either alone or associated with AD [3]. Furthermore, atherosclerosis is evidenced in cerebral arteries in AD patients [4]. Vascular risk factors (hypertension, obesity, and diabetes mellitus) are associated with an elevated dementia and amyloid overproduction risks [5,6]. A frequent comorbidity of cerebrovascular and AD pathologies is confirmed in aged subjects [7,8,9]. At the mechanistic level, a plethora of tissue and molecular events have been proposed to interplay between the neurodegenerative process and the cerebrovascular damage (blood–brain barrier leakage, inflammation, oxidative stress) [10,11], however, the complete knowledge of this potential cause-and-effect relation is still lacking [10].

Although AD can be frequently diagnosed with a considerable accuracy, the distinction between MixD, isolated AD and VaD remains controversial, being a difficult diagnostic challenge [11].

Particularly relevant to neurologists is the fact that olfactory dysfunction can be considered a clinical, or in some cases a preclinical, sign of different dementias like AD and VaD [12,13]. Although a score below normal performance in olfactory test has been observed in VaD patients [14], further sensorial studies with larger and longitudinal cohorts are necessary to evaluate potential differences in the olfactory performance between AD and VaD [14,15]. It is important to note that olfactory dysfunction has been associated with increased mortality from neurodegenerative and cardiovascular diseases [16]. Several studies point out that cardiovascular and cerebrovascular disease, subclinical atherosclerosis, stroke, and diabetes are considered predictors of accelerated odor identification decline [17,18,19]. Ischemic or structural damages in brain areas involved in olfaction have been proposed as potential drivers of this olfactory decline [20].

Given the global prevalence of MixD-associated cognitive impairment and the lack of therapeutic strategies, there is a clear unmet need for vascular therapies targeting mechanisms that precipitate the neurodegenerative process. It is well known that the molecular homeostasis of olfactory structures is deeply altered in the context of AD pathology [21,22,23,24]. However, the impact of MixD on olfactory areas remains to be clarified. In this study, we have applied an olfactory proteotyping strategy [25] to partially reveal the missing relationships in the pathobiochemical knowledge when AD and vascular damage coexist, deciphering common and differential olfactory protein mediators between pure AD and MixD. Moreover, we have used olfactory neuroproteomic data as a strategy to define potential fluid biomarkers for the diagnosis and discrimination of patients affected by AD and MixD.

## 2. Materials and Methods

### 2.1. Materials

The following reagents and materials were used: The antibodies for pMEK (#9154), MEK (#9126), pERK (#4370), ERK (#9102), pPDK1 (#3061), PDK1 (#3062), pPKCpan (#9379), pAKT (#4060), AKT (#4685), pSEK (#9156), SEK (#9152), pSAPK (#9255), SAPK (#9252), pPKAc (#5661), PKAc (#4782), pP38 (#9211), P38 (#9212), pNFκB (#3033), NFκB (#8242) were purchased from Cell Signaling. Antibody for PKCpan (SAB4502356) was purchased from Sigma Aldrich. The antibody for pPI3K (PA5-104853) was purchased from Life Technologies. Antibody for PI3K (ab86714) was purchased from abcam. Electrophoresis reagents were purchased from Biorad and trypsin from Promega.

### 2.2. Human Samples

According to the Spanish Law 14/2007 of Biomedical Research, inform written consent forms of the Brain Bank of IDIBAPS (Barcelona, Spain) were obtained for research purposes from relatives of patients included in this study. Post-mortem fresh-frozen olfactory bulbs of 6 Mixed dementia (MixD) patients, and 7 controls were obtained from the Brain Bank of IDIBAPS (Barcelona, Spain) following the guidelines of Spanish legislation. The control group was composed of elderly subjects with no histological findings of any neurological disease. The study was conducted in accordance with the Declaration of Helsinki and all assessments, post-mortem evaluations, and procedures were previously approved by the Local Clinical Ethics Committee (PI_2019/108). All human brains considered in the proteomics and follow-up phases had a post-mortem interval (PMI) lower than 19 h (Table 1). On the other hand, in order to check potential disease biomarkers, serum samples from MixD (*n* = 19) and AD (*n* = 31) patients together with serum samples from healthy subjects (*n* = 32) were collected (Supplementary Table S1). In all cases, neuropathological assessment was performed according to standardized neuropathological guidelines [26,27,28,29].

### 2.3. Olfactory Proteomics

Whole OB specimens (70–80 mg) derived from controls and MixD cases were homogenized in lysis buffer containing 7 M urea, 2 M thiourea, 50 mM DTT. After ultracentrifugation, protein extracts were precipitated, pellets were dissolved in 6 M Urea and Tris 100 mM pH 7.8 and Bradford assay kit (Bio-Rad) was used for protein quantitation. Whole proteomes were concentrated in the stacking/resolving SDS-PAGE gel interface. After staining, protein digestion (10 ug) was carried out with trypsin (Promega; 1:20, *w*/*w*) at 37 °C for 16 h as previously described [30]. Prior to LC-MS/MS, peptides were purified and concentrated using C18 Zip Tip Solid Phase Extraction (Millipore, Burlington, MA, USA). Label free LC-MS/MS analyses were performed on an EASY-nLC 1200 liquid chromatography system interfaced with a Q Exactive HF-X mass spectrometer (Thermo Scientific, Waltham, MA, USA). Chromatographic/elution conditions and mass-spectrometry parameters were as previously described [31]. Data were acquired using Xcalibur software (Thermo Scientific, Waltham, MA, USA).

### 2.4. Data Analysis

Mass spectrometry raw data were processed using the MaxQuant software (v.1.6.3.3) (Max Planck Institute, Munich, Germany) [32] following the next parameters: (1) Homo Sapiens UniProtKB database (February 2019) containing contaminants and the reversed version of all sequences, (2) main peptide search (4.5 ppm) and first search tolerance (20 ppm), (3) trypsin digestion with a maximum of two missed cleavages, (4) variable modifications (methionine oxidation and N-terminal acetylation), (5) fixed modification (carbamidomethylation), (6) peptide length (7 amino acids), (7) fragment mass deviation (40 ppm) and (8) false discovery rate (FDR) for peptide spectrum match (PSM), peptide and protein identification (1%). The analysis of the Maxquant output file and subsequent visualization was done by Perseus software [33]. Potential contaminants and proteins identified as reverse were removed. The data were transformed into log2 values and normalization was performed using a width adjustment strategy. Protein identification and quantitation criteria was performed as previously described [31]. The protein identification was considered valid with at least two unique or razor peptides whereas protein quantification was calculated using at least two unique peptides. For differential analysis, a 1.3-fold change cut-off was used (two-way Student *T*-test; *p* < 0.05). Hence, proteins with ratios below the low range of 0.77 were considered downregulated whereas those with higher range than 1.33 were considered up-regulated. MS data and search results files were deposited in the Proteome Xchange Consortium via the JPOST partner repository (https://repository.jpostdb.org, accessed on 13 April 2021) [34] with the identifier PXD025368 for ProteomeXchange and JPST001128 for jPOST. Interactome and pathway analysis were performed using BioGrid [35], Ingenuity (Qiagen), or Metascape [36] tools.

### 2.5. Western-Blotting

Equal amounts of OB protein (10 µg) were resolved in 4–15% stain free SDS-PAGE gels (Bio-rad) and electrophoretically transferred using a Trans-blot Turbo transfer system (up to 25 V, 7 min) (Bio-rad). Membrane probing, immunoreactivity visualization, equal loading, digitalization, and densitometric analysis was performed as previously described [31].

### 2.6. Enzyme-Linked Immunoabsorbent Assay

Serum Neurexin-3 (NRXN3), Tenascin-R (TNR) and Prolow-density lipoprotein receptor-related protein 1 (LRP1) concentrations were measured using enzyme-linked immunoabsorbent assay (ELISA) kits according to the manufacturer’s instructions (MBS93337537; MBS728632; MBS2021100-Mybiosource). The data were analyzed using Graphpad Prism Software and Mann–Whitney U test was used to make group comparisons. A *p*-value less than 0.05 was considered statistically significant.

## 3. Results and Discussion

Unbiased omics approaches have been proposed as essential tools to increase our understanding of the AD pathogenesis subtype variety as well as the common presence of vascular effects present in mixed pathologies [37]. Specifically, proteomics has already been aimed to provide more insights into VaD at cortical level [38,39]. Although it is widely known that patients suffering from AD and VaD experience olfactory dysfunction [12,13], no studies have examined the impact of this sensorial impairment at molecular level.

### 3.1. Olfactory Bulb Proteome-Wide Characterization in Human MixD

Since olfactory system is considered a potential gateway for the access of environmental insults and a prion-like propagation site in different forms of dementia [40,41], we have used OB label-free quantitative proteomics to deeply characterize the olfactory proteostatic imbalance in MixD (Table 1).

**Figure 1:**
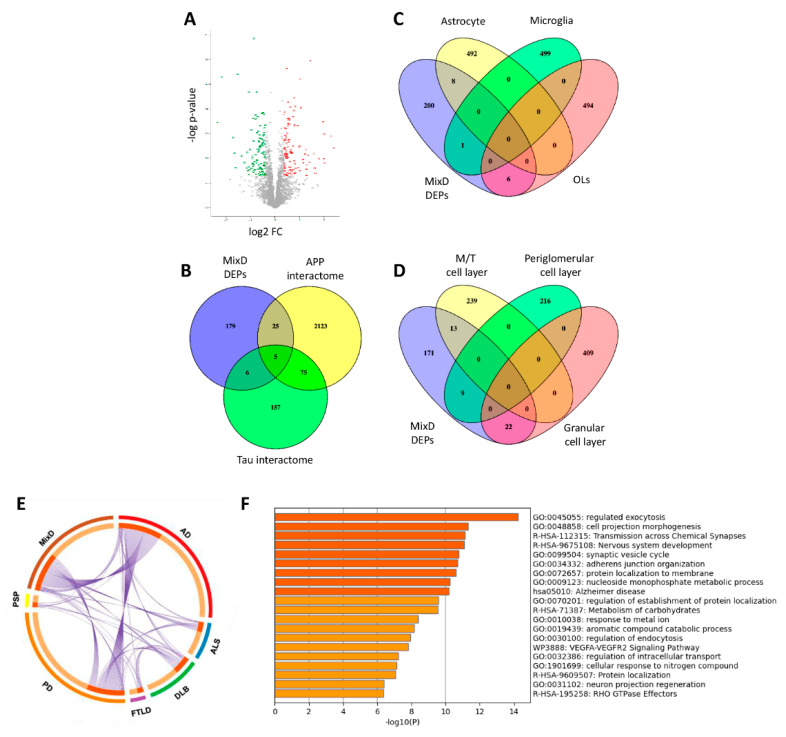
Olfactory proteome-wide analysis in MixD. (**A**) Volcano plot indicating the statistically significant DEPs represented in red (upregulation) and green (downregulation). (**B**) Venn diagram showing the overlap between OB DEPs and experimentally demonstrated APP and Tau interactors. (**C**) Cluster-enriched genes in specific brain cells that are differentially expressed in the OB from MixD subjects. (**D**) Cluster-enriched genes in specific OB cell layers that are deregulated in MixD at the level of the OB. (**E**) Circos plot representing commonalities in DEPs (purple lines) between MixD and different neurological phenotypes: AD, Parkinson’s disease (PD), Dementia with Lewy Bodies (DLB), Progressive supranuclear palsy (PSP), Amyotrophic lateral sclerosis (ALS), frontotempral lobar degeneration TDP-43 proteinopathy (FTLD-TDP43). (**F**) Top-20 statistically enriched terms from MixD DEPs generated by Metascape.

Among 4572 identified proteins, 2440 proteins were quantified across all samples (Supplementary Table S2), from which 215 proteins were differentially expressed (DEPs) in OBs derived from MixD subjects compared to neurological intact controls (107 upregulated and 108 downregulated proteins in MixD) (Figure 1A, Supplementary Table S2, Supplementary Figure S1). Based on the information stored in the BIOGRID repository [35], a subset of DEPs were experimentally demonstrated to be components of the interactomes of typical neuropathological substrates found in AD. As shown in Figure 1B, 25 DEPs belong to the amyloid precursor protein (APP) interactome (*SNAP25*, *PPP3CC*, *NDUFS4*, *GORASP2*, *GFAP*, *SGIP1*, *COX5B*, *DMTN*, *CAMKV*, *EEF1B2*, *UQCRB*, *PSMC1*, *GNAZ*, *PLCD1*, *PSAT1*, *APRT*, *AK1*, *SH3BGRL3*, *PLIN3*, *PPIA*, *MAT2B*, *CSRP1*, *MGST3*, *ARL8B*, *LRP1*) whereas 6 DEPs (*YBX1*, *FUS*, *PTK2B*, *EZR*, *DENR*, *S100B*) corresponds to Tau interactors. Five DEPs are part of the shared interactome between APP and Tau (*SRC, PRKCG*, *SLC25A4*, *MAPT*, *MAPRE1*). Due to our olfactory proteotyping workflow is not able to differentiate specific cell layers, we have performed cell-specific enrichment analysis across olfactory DEPs using public single-cell RNA-seq data [42,43]. As shown in Figure 1C, 8 DEPs (*GFAP*, *EZR*, *GPT*, *ENTPD2*, *MLC1*, *FABP1*, *TNC*, *MACF1*) are considered highly enriched genes in astrocytes, PTK2B in microglia and *COL1A2*, *ACAN*, *ITGB8*, *RCN1*, *DPP6*, *S100A1* in oligodendrocytes. In addition, part of the differential proteome tend to be specifically enriched in OB mitral/tufted cell layers (*SCG2*, *CNTNAP2*, *THY1, RCN2*, *FUS*, *PAM*, *VSNL1*, *RTN3*, *ERC2*, *REEP5*, *NBEA*, *SV2A*, *MACF1*), periglomerular cell layer (*GAP43*, *LYH6*, *HPCAL1*, *CD200*, *UQCRB*, *PDE2A*, *SLC25A4*, *ENO1*, *MPC2*) and granular cell layer (*BASP1*, *YBX1*, *CALM2*, *SNAP25*, *OPCML*, *NRXN3*, *SGIP1*, *CAMKV*, *DYNC1I1*, *ATP6V1G2*, *NEGR1*, *CADM3*, *PTK2B*, *VBP1*, *ICAM5*, *CACNA2D1*, *PHPT1*, *SH3BGRL3*, *PFN2*, *MGST3*, *SF3B1*, *HMGB1*) (Figure 1D). All these data shed new light about the molecular disturbances that are involved in the olfactory neurodegeneration across each cell-type homeostasis in MixD. Our analysis also revealed that part of the DEPs are not specific of MixD (Figure 1E), being common OB deregulated proteins previously observed across different tauopathies, synucleinopathies, and tardopathies [23,24,31,44,45]. Functional cluster analysis of the DEPs found in MixD group reveal an enrichment in either neuron-specific or neuron-relevant processes (Figure 1F).

### 3.2. MixD Induces Olfactory Disruption in Functional Tau/APP Interactomes and Specific Survival Pathways

**Figure 2:**
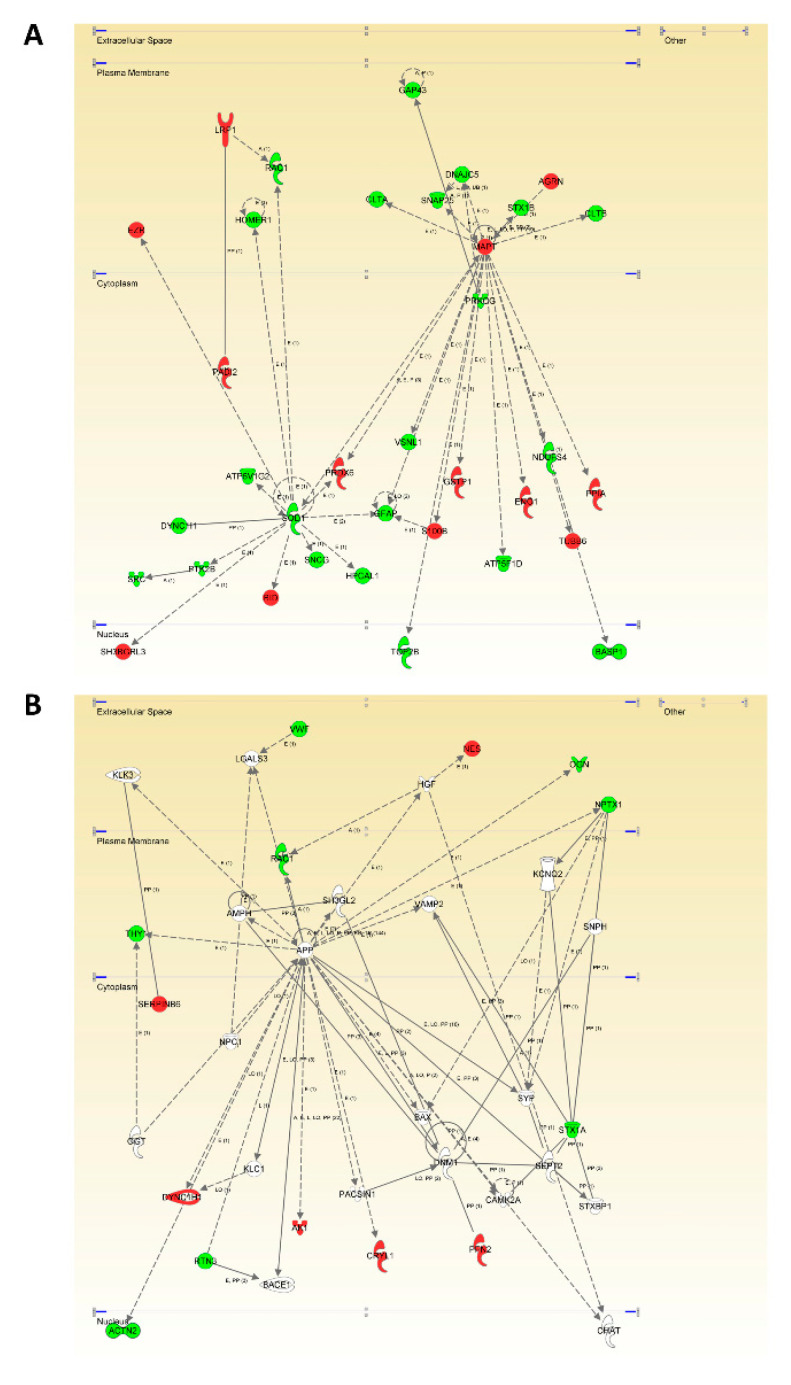
OB Modulation of the MAPT (**A**) and APP (**B**) functional interactomes in MixD. Relationships between DEPs and potential neuropathological substrates are represented by continuous lines (direct interactions) or discontinuous lines (indirect functional interactions). Up-regulated and down-regulated proteins are marked in red and green, respectively (for complete legend interpretation, see Supplementary File S1.

Bearing in mind that the characterization of unexpected connections between seemingly unrelated proteins and neuropathological substrates is a straightforward approach for the identification of novel MixD related-targets, we explored whether Tau (*MAPT*) and APP were functionally interconnected with DEPs in MixD OBs. Proteome-scale interactome maps merging the OB DEPs were performed using the IPA software (Figure 2 and Supplementary File S1). Interestingly, 20 differential functional interactors for Tau were identified, suggesting the involvement in related biological functions. Specifically, olfactory Tau is central to an interconnected molecular network between plasma membrane (*CLTA*, *SNAP25*, *DNAJC5*, *STX1B*, *AGRN*, *CLTB*) and nucleocytoplasmic region (*PRKCG*, *PPIA*, *NDUFS4*, *TUBB6*, *BASP1*, *ENO1*, *ATP5F1D*, *GSTP1*, *S100B*, *TOP2B*, *VSNL1*, *GFAP*, *PRDX6*, *SOD1*). However, the deregulated olfactory APP interactome impacts across extracellular space (*VWF*, *NES*, *OGN*, *NPTX1*), plasma membrane (*THY1*, *RAC1*), and cytoplasm (*SERPINB6*, *DYNC1H*, *RTN3*, *AK1*, *CRYL1*, *PFN2)* (Figure 2).

**Figure 3:**
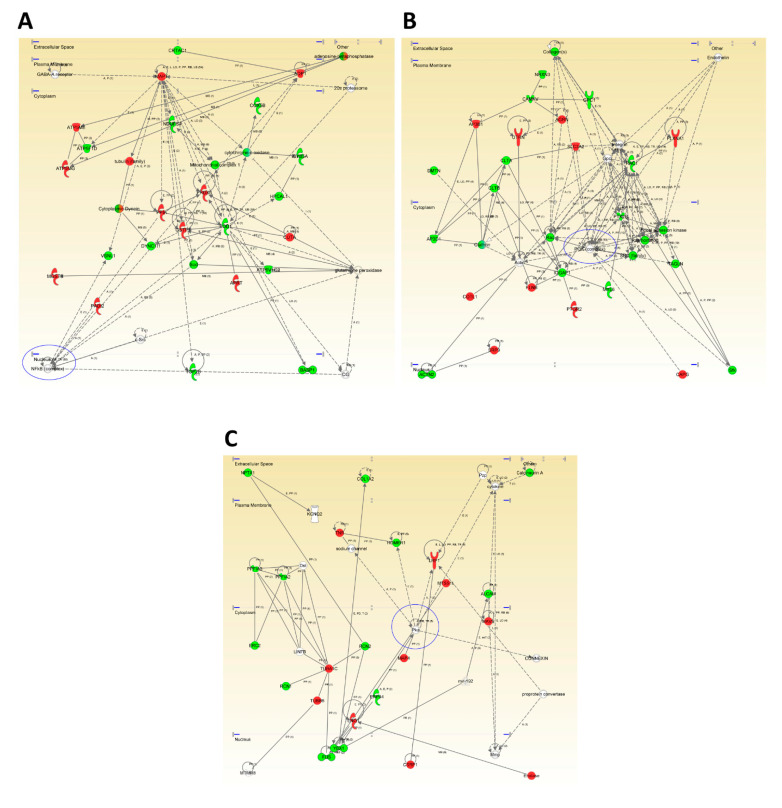
High/scoring protein interactome networks for differential expressed proteins in the OB of MixD subjects. (**A**–**C**) show hubs proposed by IPA software focused in NFκB (**A**), Pi3K (**B**) and PKA (**C**). Up-regulated and down-regulated.

**Figure 4:**
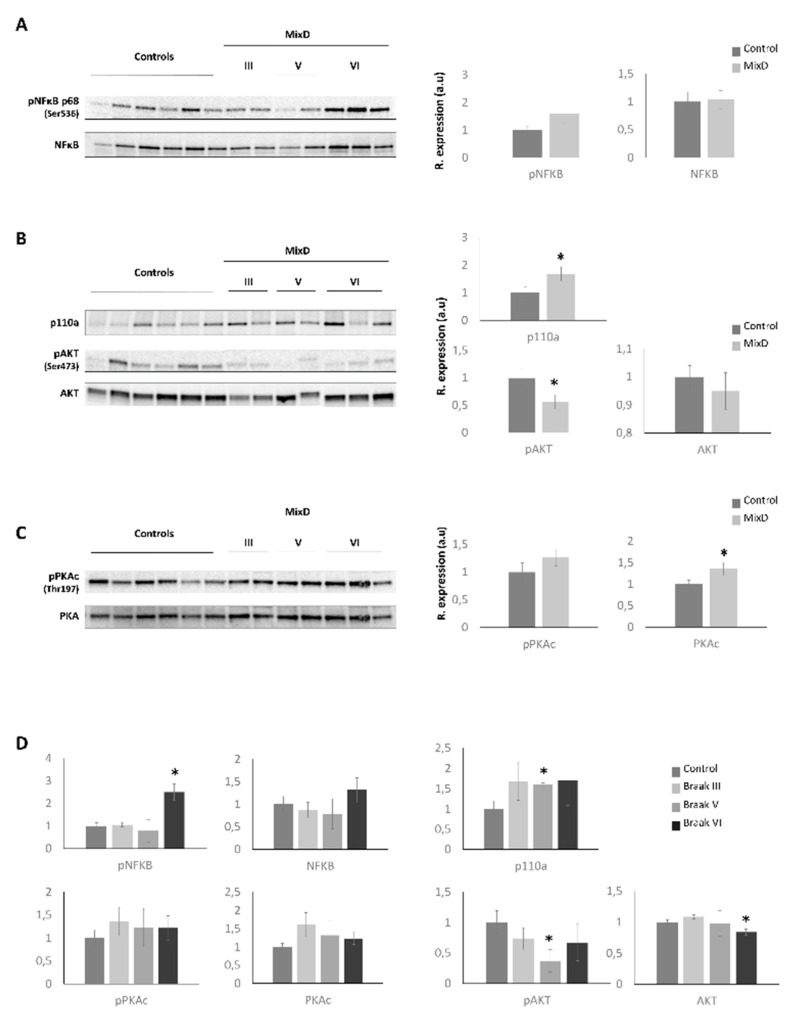
Monitoring of the specific signaling modulators that appeared as principal nodes in our protein interactome maps. Levels and phosphorylation of NFκB (**A**), p110a, AKT (**B**), and PKAc (**C**) were analyzed across MixD OB samples. Analysis was also performed separating MixD group by Braak staging (**D**), (* *p* < 0.05 versus control group).

In a wider scale, a whole proteome comparison revealed that signaling mediators like the nuclear factor kappa B (NFκB) and PI3K complexes and cell-stress related such as PKA appeared as principal hubs in functional interactome maps (Figure 3, Supplementary File S1). As shown in Figure 3A, the deregulation of several mitochondrial-related proteins (*ATP5ME*, *ATP5F1D*, *ATP5MG*, *NDUFS4*, *COX5B*, *Cytochrome c oxidase, CYB5A*, *SOD1*, *GSTP1*) suggested an impairment in mitochondrial function in the OB of MixD subjects. In accordance, mitochondrial dysfunction constitutes an early and well-known feature of neurodegenerative processes [46] and our group has previously described alterations in the mitochondrial sensor PHB complex across several-related neurological disorders, including MixD [23]. On the other hand, although the NFκB constitutes a master regulator of many essential signaling cascades when activated travelling from the cytoplasm to the nucleus [47], the recently described presence of mitochondrial NFκB suggest its influence on important mitochondrial processes [48]. Therefore, subsequent experiments were performed in order to study the activated status on NFκB. As shown in Figure 4A,D, although no significant changes were found when analyzing all the study samples at the same time, a significant increase in the activated levels of NFκB was observed in subjects diagnosed with the highest Braak stages (Braak VI). Of note, NFκB role on cell survival can be either neuroprotective or induce neurotoxicity by proinflammatory mechanisms. Depending on the pathological state, its overexpression can result in damage to the vessel walls and impaired vascular cell function [49]. On the other hand, in order to enhance the analytical outcome of our proteomic experiment, the activated status of the PI3K complex and the cAMP-dependent protein kinase A (PKA) was also monitored (Figure 3B,C, respectively). As shown in Figure 4B,D, although a slight upregulation of p110a (PI3K catalytic subunit) protein levels was evidenced, more predominantly in Braak V stages, an AKT inactivation was observed, suggesting a potential role of phosphatases such as PP2A, PTEN, or others in this context [50]. Likewise, the PI3K/Akt signaling pathway mediates cell survival and differentiation, and participates in learning and memory processes [51]. Interestingly, significant changes in AKT levels were not detected in the OB of AD subjects [52], suggesting that the vascular damage may be responsible for this deregulation at the level of the OB. On the contrary, significant increased levels of the catalytical subunit of PKA (PKAc) were found (Figure 4C), where the tendency was mainly observed in the Braak III stages (Figure 4D), thus, indicating alterations in cAMP levels. In this sense, similar alterations in AD subjects have been observed, suggesting common OB molecular mechanisms between these two pathologies.

### 3.3. Comparison of OB Deregulated Proteome in Pure AD and Mixed Dementia: An Specificity Analysis

To further study in detail the OB metabolic modulation in MixD, the differential proteome map was functionally characterized. As shown in Figure 1F, synaptic transmission, nucleoside monophosphate metabolism, carbohydrate metabolism, response to metal ion, aromatic catabolism, intracellular transport, neuron projection regeneration and VEGF signaling were part of the most significantly overrepresented bioprocesses in MixD subjects (Supplementary Table S2 and Supplementary Figure S2). Bioinformatic analysis also revealed that a subset of OB proteins was linked to AD and/or vascular processes such as blood vessel development, atherosclerosis, and coagulation (Table 2).

**Figure 5:**
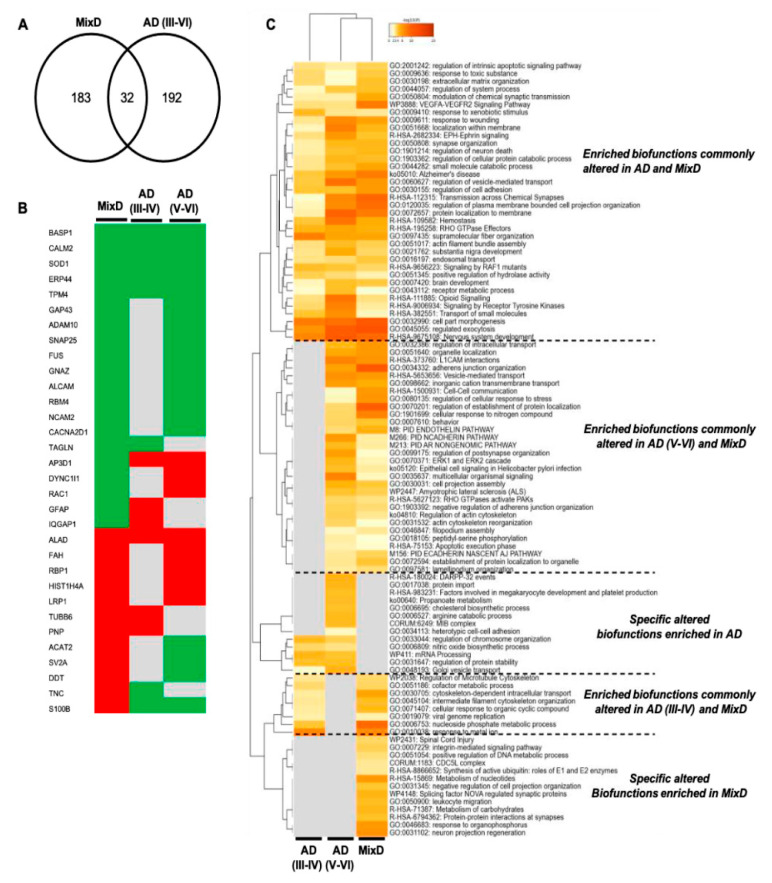
Comparative analysis between differential OB proteomes across AD and MixD. (**A**) Venn diagram showing the overlap od DEPs detected in AD (Braak stages III-VI) and MixD phenotypes. (**B**) OB Protein expression profile of the 32 common deregulated proteins between AD and MixD (green: downregulated; red: upregulated; grey: not differentially expressed). (**C**) Commonalities and differences observed in the significantly altered functional profile between AD and MixD phenotypes.

In order to partially decipher unspecific and specific proteostatic alterations due to the presence of concomitant AD, we interlocked our MixD differential dataset with OB differential proteome data previously obtained from pure AD cases [23]. Due to the MixD samples used in our proteomics workflow derived from subjects with concomitant AD (Braak stages III-VI), only differential proteins detected across these Braak stages in our previous work were considered. As shown in Figure 5A, 32 protein mediators were deregulated not only in MixD but also in pure AD. However, only the protein expression profile corresponding to 6 proteins (*BASP1*, *CALM*, *SOD1*, *ERP44*, *TPM4*, *ALAD*) was similar across AD and MixD OBs (Figure 5B). Interestingly, functional clustering unveiled common and distinct imbalanced biological processes between MixD and AD (Figure 5C).

**Figure 6:**
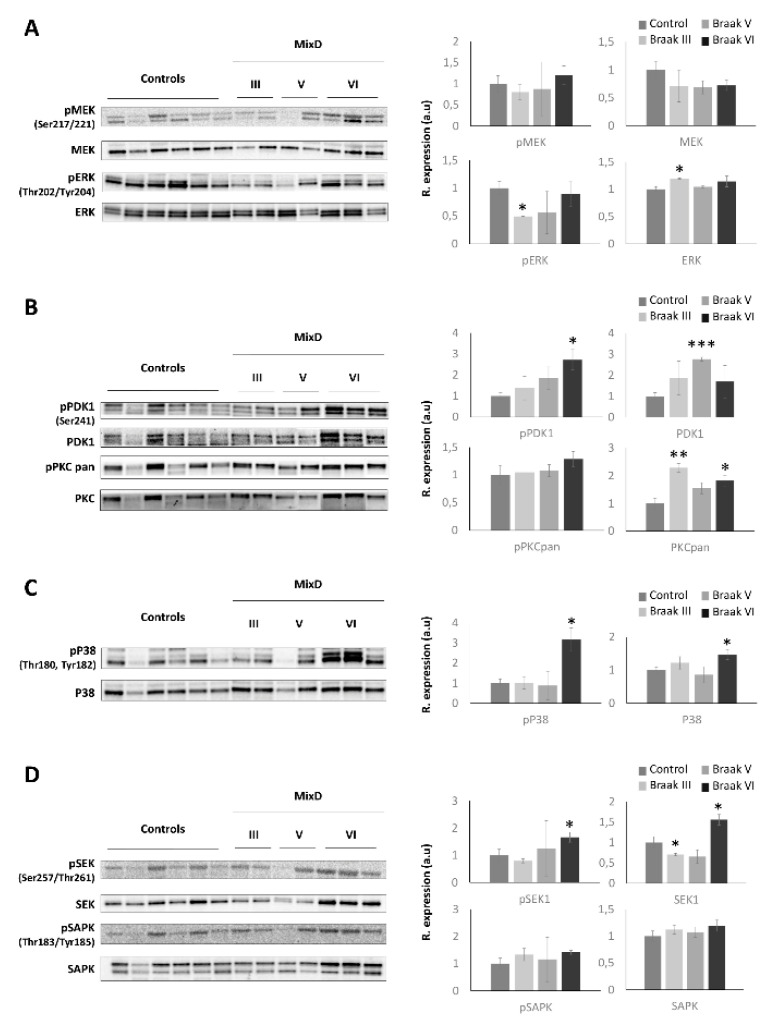
Olfactory expression of survival routes across MixD stages. Levels and phosphorylation of MAP kinases (**A**), PDK1-PKC (**B**), p38 MAPK (**C**), and SEK1-SAPK (**D**). * *p* < 0.05 versus control group; ** *p* < 0.01 versus control group; *** *p* < 0.001 versus control group.

Specifically, DEPs that map in functional categories such as integrin-mediated signaling pathway, CDC5L complex, regulation of cell projection organization, leukocyte migration, synaptic protein-protein interactions and neuron projection regeneration were exclusively and significantly enriched in MixD (Figure 5 and Supplementary Tables S3 and S4). These data indicate that the comparative analysis between omics outputs may be considered a useful tool to potentially distinguish pure AD and MixD pathologies through the elucidation of specific biological process as well as the identification of potential discriminatory biomarkers. On the other hand, other biological pathways were commonly deregulated between the two correlated pathologies (Figure 5C). Among them, cell-stress related pathways were potentially deregulated according to the differential proteomic signature in both AD and MixD. Therefore, we decided to monitor a kinase panel gathering essential biological pathways due to (i) previous findings showing alterations in cell-survival and stress related pathways across different neurodegenerative disorders at the level of the OB [24,44,45], and (ii) the absence of similar reports focused on MixD contexts. First, regarding the MAPK pathway, a significant decrease in the activated levels of ERK ½ was observed in the MixD cases diagnosed with Braak III staging (Figure 6A and Supplementary Figure S3), interestingly opposite to the hyperactivation previously observed in the OB of AD subjects [23]. Concerning the PDK1-PKC axis, a significant increase in the activated levels of PDK1 was observed in the Braak V stages, just as an increment in the total levels of the PKC family in the Braak III and VI staging (Figure 6B and Supplementary Figure S3). Deregulations in the PKC signaling cascades are known to be early features in the brain of patients with AD [53] and previous reports in olfactory AD samples have also reported stage-specific deregulations in this axis [23]. However, to our knowledge, this is the first report linking alterations in this route in MixD backgrounds. On the other hand, since the p38 MAPK signaling has been extensively linked to neurodegeneration and inflammatory processes [54,55], we further evaluated the activated status of p38 MAPK in MixD OBs. As shown in Figure 6C and Supplementary Figure S3, both activated and total levels of P38 were upregulated but only in Braak VI stages, suggesting a neuroinflammatory environment at the level of the OB. In line with this findings, recent studies have shown that the activation of this pathway may lead neuronal apoptosis and functional deficits in vascular dementia [56]. In accordance, stage-specific alterations were also found for both activated and total levels of SEK1, again demonstrating altered cell-stress responses at olfactory level during MixD disorders.

### 3.4. Protein Serum Profile Across AD and MixD: A Pilot Study Targeted to the Analysis of Neurexin-3 (NRXN3), Tenascin-R (TNR) and Prolow-Density Lipoprotein Receptor-Related Protein 1 (LRP1)

**Figure 7:**
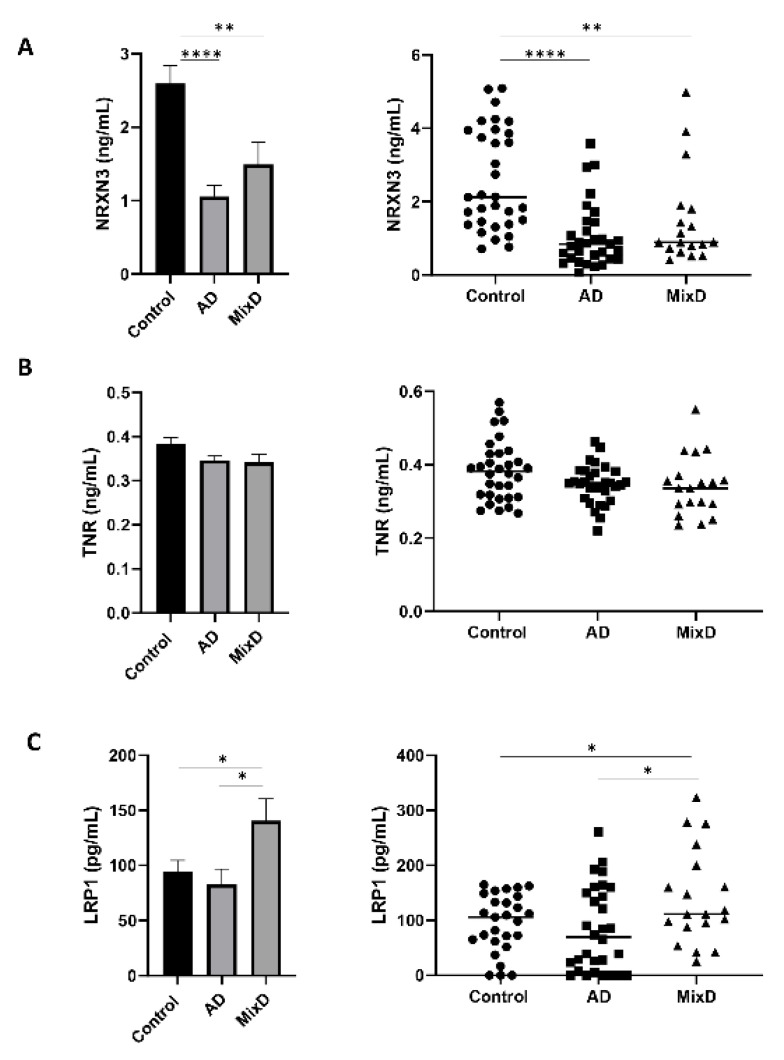
Monitoring of NRXN3 (**A**), TNR (**B**), and LRP1 (**C**) protein levels across serum samples from AD and MixD subjects. Protein levels were measured in sera derived from 82 individuals (30 controls; mean age: 70 years; 15 F/15 M), 30 patients with AD: mean age 75 years, 15 F/15 M, and 19 patients with MixD: mean age 79 years; 10 F/9 M). * *p* < 0.05; ** *p* < 0.01; **** *p* < 0.0001.

More than half of the patients meeting clinicopathological AD diagnostic criteria also have vascular lesions [3]. Based on the neuropathological co-existence and the controversy regarding the differential diagnosis between AD and MixD, there is an urgent need for a better clinical differentiation of these pathologies. Bearing in mind that fluid proteomics is considered a valuable molecular repository for diagnosing/targeting the neurodegenerative process and olfactory neurodegeneration is among the earliest features, the application of olfactory proteomics is an ideal bridge to detect olfactory proteins that might be tested in fluids as potential biomarkers [57]. Aiming to discover potential biomarkers to differentiate neurological syndromes, we have focused our attention on three proteins (NRXN3, TNR, LRP1) because they are tentatively present in biofluids and are involved in exclusive altered biofunctions enriched in MixD (Supplementary Table S3). Specifically, NRXN3 is related to cognition, learning/memory (Table 2) and protein–protein interactions at synapses (R-HSA-6794362; Figure 5C); TNR is also related to cognition, learning/memory (Table 2) and regulation of cell projection organization (GO: 0031345; Figure 5C); and LRP1 is related to AD, blood vessel development (Table 2) and neuron projection regeneration (GO:00311102; Figure 5C). For that, serum samples belonging to AD and MixD phenotypes together with non-neurological controls were included in the study (*n* = 32/control; *n* = 31/AD; *n* = 19/MixD; ~50/50 female/male) (Supplementary Table S1). To our knowledge, no experimental evidence have linked NRXN3, TNR, and LRP1 with MixD. Alterations in presynaptic adhesion NRXN3 protein levels have been linked with a major AD risk [58]. Interestingly, a significant decrease in NRXN3 serum levels was observed between both AD and MixD samples (Figure 7A) and neurological intact controls. However, although our data suggest a more prominent decrease in NXRN3 serum levels in AD, no significant changes were observed between both pathologies. On the other hand, while serum TNR protein levels were unaltered between neurological contexts and healthy controls (Figure 7B), a significant increase in LRP1 serum protein levels was observed in MixD subjects maintaining normal levels in AD (Figure 7C). In this sense, although an effort to find specific sex differences was performed analyzing our data (Supplementary Figure S4), no significant changes were observed for any of the biomarkers. In particular, LRP1 is an ApoE receptor that plays a role in clearance of Abeta and regulates glucose uptake and insulin signaling in the brain, being a key regulator of Tau uptake and spread [59,60,61]. In this sense, being aware of the limited cohort analyzed, our data indicate that LRP1 may be a potential biomarker able to distinguish between both syndromes. Raw quantifications are shown in Supplementary Table S5. These results should be further evaluated in larger cohorts and in combination with other biochemical markers in order to improve the current diagnostic assays.

Although this study has uncovered many intricacies in the OB homeostasis in the context of MixD, there are potential limitations that warrant discussion. Due to the technological approach used, we failed to accurately monitor many protein species with low expression levels. Both AD pathology and cerebrovascular disease independently are strongly related with cognitive decline and/or dementia. Frequently, they appear together, showing an additive or even a synergistic effect and the weight of each component may be different among patients. In those cases, it remains challenging to distinguish between AD and MixD. This difficulty in diagnosis limits the number of MixD patients available in our cohort. Furthermore, even in absence of cognitive impairment, it is difficult to find pure age-matched controls without any sign of amyloid pathology or cerebrovascular disease given the high incidence of these lesions in the elderly and the fact that both findings increase with age. This factor is the reason why our control group has decreased age compared with MixD group. Therefore, we could not exclude the possibility that part of the differences found between both groups could be influenced by other age-related factors apart from the MixD ocurrence. Regarding the vascular component of MixD, different cerebrovascular lesions and locations induce different phenotypes of dementia. Given a big enough cohort of subjects, OB proteomics may prove to be useful to discriminate different types of dementia according to different cerebrovascular lesions.

## 4. Conclusions

Overall, the present study provides new clues regarding the molecular mechanisms concerning the olfactory dysfunction that occurs during MixD. Part of the differential OB protein set was not specific of MixD, being also deregulated across different tauopathies, synucleinopathies and tardopathies. However, functional analysis has unveiled OB commonalities and differences between pure AD and MixD. Based on olfactory proteomic data, LRP1 may be considered a potential serum biomarker to differentiate AD and MixD phenotypes.

## Figures and Tables

**Table 1:** OB samples from MixD patients and controls. F (female), M (male), PMI (post-mortem interval), WB (Western Blot).

Groups	Sample	Age (years)	Sex	PMI	Neuropathological Diagnosis	Proteomics	WB
Control	1218	65	F	3 h 45 m	AD I/0	+	-
	1277	74	M	9 h 25 m	AD I/0	+	+
	1368	45	M	18 h 30 m	AD 0/0	+	+
	1403	51	F	4 h	AD 0/0	+	+
	1438	67	M	5 h 50 m	AD I/0, Amyloid angiopathy	+	+
	1563	59	F	5 h 30 m	AD I/0, Metastatic carcinoma	+	+
	1576	60	F	12 h	AD I/0	+	+
MixD	DM4	81	F	9 h 40 m	AD III/B, vascular encephalopaty	+	+
	DM5	89	M	11 h 30 m	AD III/A, vascular encephalopathy	+	+
	DM6	85	M	12 h	AD V/B, vascular pathology	-	+
	DM8	88	F	5 h	AD V/B, vascular pathology	+	+
	DM1	83	M	2 h 30 m	AD VI/C, infarcts	+	+
	DM3	76	M	6 h 30 m	AD VI/C, infarcts	+	+
	DM7	81	M	15 h	AD VI/C, infarcts	+	+

**Table 2:** OB DEPs in MixD previously related with AD and/or vascular damage. FC; fold change. Unique peptides correspond to the number of exclusive peptides that have been used for the protein quantitation of each protein. The biological involvement information derived from the Metascape output files.

Protein Names	Gene Name	FC	*p* Value	Unique Peptides	Biological Involvement
Collagen alpha-2(I) chain	COL1A2	0.20	0.000	4	blood vessel development, interactions at the vascular wall, coagulation
Cadherin-13	CDH13	0.32	0.006	3	cardiovascular & blood vessel development
G protein-regulated inducer of neurite outgrowth 1	GPRIN1	0.39	0.008	24	AD
Calmodulin	CALM	0.45	0.006	12	AD, VEGFR2 mediated vascular permeability, atherosclerosis
Disintegrin and metalloproteinase domain-containing protein 10	ADAM10	0.48	0.022	10	AD, cardiovascular development
Synaptosomal-associated protein 25	SNAP25	0.48	0.000	16	Cognition, learning/memory
Secretogranin-2	SCG2	0.54	0.021	15	cardiovascular & blood vessel development
Proto-oncogene tyrosine-protein kinase Src	SRC	0.54	0.017	5	interactions at the vascular wall, atherosclerosis, coagulation
Serine/threonine-protein phosphatase 2B catalytic subunit gamma	PPP3CC	0.54	0.015	4	AD
NADH dehydrogenase [ubiquinone] iron-sulfur protein 4, mitochondrial	NDUFS4	0.54	0.002	6	AD
Integrin beta-8	ITGB8	0.56	0.014	3	cardiovascular & blood vessel development
Neurexin-3	NRXN3	0.58	0.000	18	Cognition, learning/memory
Contactin-associated protein-like 2	CNTNAP2	0.58	0.007	12	Cognition, learning/memory
Thy-1 membrane glycoprotein	THY1	0.62	0.005	4	cardiovascular & blood vessel development
Rap guanine nucleotide exchange factor 2	RAPGEF2	0.63	0.043	11	cardiovascular & blood vessel development
Cytochrome c oxidase subunit 5B, mitochondrial	COX5B	0.63	0.002	6	AD
Protein kinase C gamma type	PRKCG	0.63	0.029	23	Cognition, learning/memory, coagulation
Dematin	DMTN	0.65	0.023	10	coagulation
Glypican-1	GPC1	0.65	0.048	9	interactions at the vascular wall, atherosclerosis
ATP synthase subunit delta, mitochondrial	ATP5D	0.67	0.025	5	AD
von Willebrand factor	VWF	0.68	0.046	11	coagulation
Tyrosine-protein phosphatase non-receptor type substrate 1	SIRPA	0.68	0.002	18	interactions at the vascular wall
Cytochrome b-c1 complex subunit 7	UQCRB	0.69	0.006	8	AD
Ras-related C3 botulinum toxin substrate 1	RAC1	0.72	0.006	4	VEGFR2 mediated vascular permeability, atherosclerosis, coagulation
cGMP-dependent 3,5-cyclic phosphodiesterase	PDE2A	0.73	0.001	26	cardiovascular & blood vessel development
Reticulon-3	RTN3	0.73	0.000	7	AD
Protein-tyrosine kinase 2-beta	PTK2B	0.74	0.049	19	cardiovascular & blood vessel development
Microtubule-associated protein tau	MAPT	1.39	0.048	8	AD, cognition, learning/memory
4F2 cell-surface antigen heavy chain	SLC3A2	1.43	0.000	28	interactions at the vascular wall
Glutathione S-transferase P	GSTP1	1.47	0.000	15	atherosclerosis
1-phosphatidylinositol 4,5-bisphosphate phosphodiesterase delta-3	PLCD3	1.55	0.030	17	cardiovascular & blood vessel development
Aquaporin-1	AQP1	1.56	0.006	3	cardiovascular & blood vessel development
Peptidyl-prolyl cis-trans isomerase A	PPIA	1.56	0.005	13	interactions at the vascular wall, coagulation
Tenascin-R	TNR	1.57	0.006	46	Cognition, learning/memory
Ectonucleoside triphosphate diphosphohydrolase 2	ENTPD2	1.60	0.040	11	coagulation
Cysteine and glycine-rich protein 1	CSRP1	1.73	0.000	12	coagulation
BH3-interacting domain death agonist	BID	1.74	0.040	5	AD
Microsomal glutathione S-transferase 3	MGST3	2.03	0.000	7	atherosclerosis
AP-3 complex subunit beta-1	AP3B1	2.45	0.019	13	coagulation
Protein S100-A1	S100A1	2.65	0.014	4	cardiovascular & blood vessel development
Cytochrome b-c1 complex subunit 9	UQCR10	2.72	0.000	2	AD
Prolow-density lipoprotein receptor-related protein 1	LRP1	3.98	0.011	37	AD, cardiovascular & blood vessel development
High mobility group protein B1	HMGB1	4.04	0.015	7	cardiovascular & blood vessel development
Protein S100-B	S100B	4.05	0.001	4	Cognition, learning/memory

## Data Availability

MS raw data and search results files were deposited in the Proteome Xchange Consortium (http://proteomecentral.proteomexchange.org, accessed on 13 April 2021) via the JPOST partner repository with the identifier PXD025368 for ProteomeXchange and JPST001128 for jPOST.

## Supplementary Materials

The following are available online at https://www.mdpi.com/article/10.3390/jpm11060503/s1, Supplementary Table S1: Samples included in the potential biomarker discovery phase monitoring serum protein levels of NXRN3, TNR and LRP1. Supplementary Table S2: OB quantified proteome and differential expressed proteins across MixD phenotypes. Supplementary Table S3: Functional analysis of OB DEPs in MixD subjects using Metascape. Supplementary Table S4: Functional clustering comparing OB DEPs in AD stages III-IV, stages V-VI and MixD subjects using Metascape. Supplementary Table S5: Raw quantification data from NXRN3, TNR and LRP1 ELISA analysis. Supplementary Figure S1: Graphical representation of the fold-change and *p*-value variables of the differential proteins. (a) Frequency histogram representation showing by means of bars the numerical data distribution of the fold-change level and (b) *p*-value of the differential proteins. Each bar represents the number of observations (frequency) on each variable in a given range. (c) Visualization of the distribution of data over a continuous interval by means of a density plot of the fold-change level and (d) *p*-value. The peaks of the density plot show where the values are concentrated in the interval. (e) Combination of histogram and density plot of the fold-change level and (f) the *p*-value. Supplementary Figure S2: Top 100 enriched ontology clusters across MixD olfactory differential proteome by Metascape. Supplementary Figure S3: Signaling pathways monitoring in the OB across MixD. Levels and residue-specific phosphorylation of MAP kinases (A), PDK1-PKC (B), P38 (C), and SSEK1.SAPK (D) in the OB across MixD phenotypes. Equal loading of the gels was assessed by total stain in each gel lane as previously described (REF) (* *p* < 0.05 versus control group; ** *p* < 0.01 versus control group). Supplementary Figure S4: Sex-specific alterations of serum NXRN3 (A), TNR (B) and LRP1 (C) protein levels across AD and MixD patients, Supplementary File S1: Legend associated to the Ingenuity Pathway Analysis software.
