# Supplementary material for: Olfactory Bulb Proteomics Reveals Widespread Proteostatic Disturbances in Mixed Dementia and Guides for Potential Serum Biomarkers to Discriminate Alzheimer Disease and Mixed Dementia Phenotypes
Source: J Pers Med. 2021 Jun 3;11(6):503. doi: 10.3390/jpm11060503 (PMC8227984; doi:10.3390/jpm11060503)
Supplement: Supplementary file 1 [file jpm-11-00503-s001.zip › SUPPLEMENTARY FIGURES AND TABLES/Supplementary File 1.pdf]

IPA Legend

Description

IPA Legend

This legend provides a key of the main features of Network Explorer and Canonical Pathways, including molecule shapes and colors as well as relationship labels and types.

Molecule Shapes

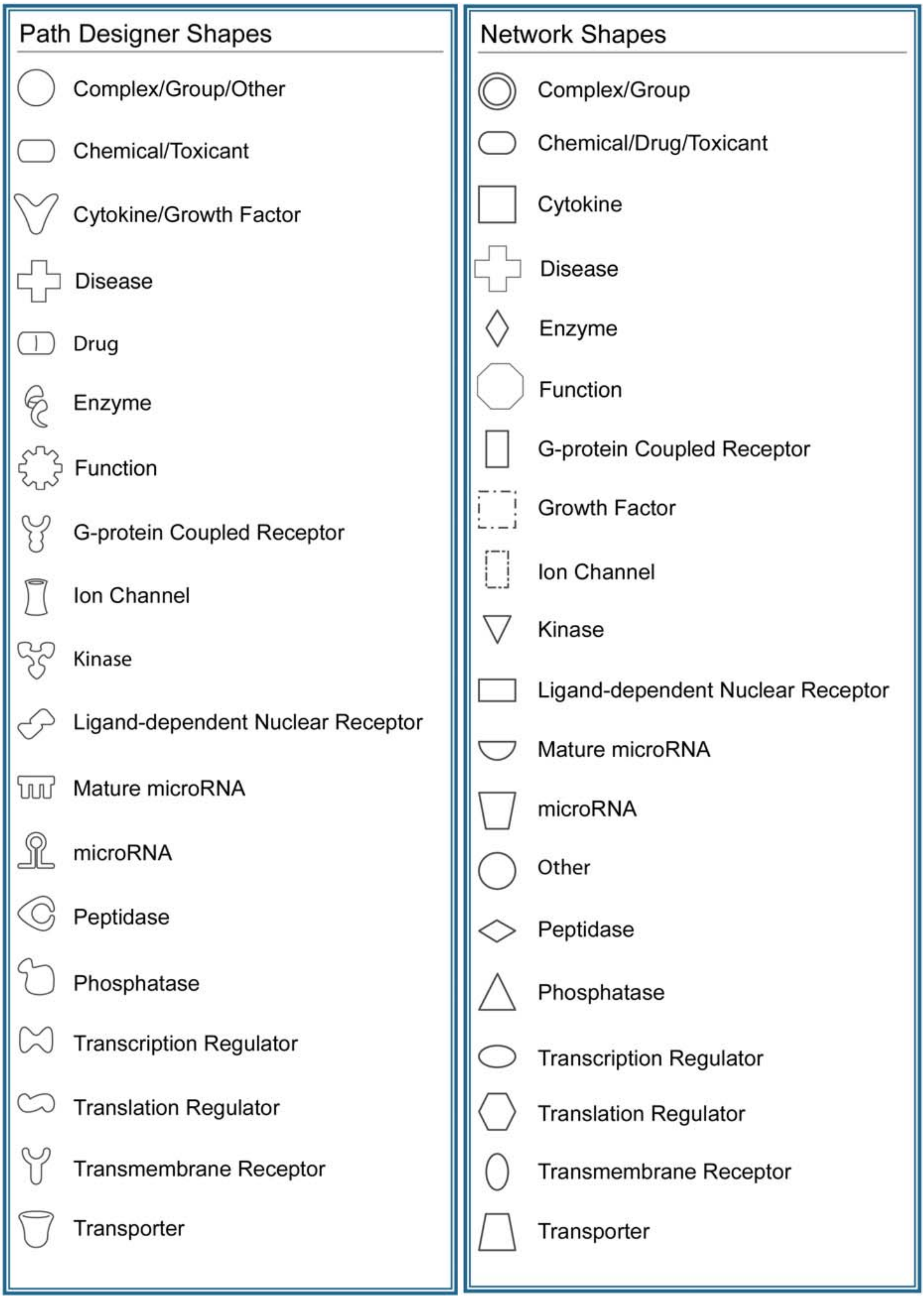

Relationship Types and Labels

Relationship Labels

A	Activation
B	Binding
C	Causation/Leads to
CO	Correlation
CC	Chemical-Chemical interaction
CP	Chemical-Protein interaction
E	Expression (includes metabolism/ synthesis for chemicals)
EC	Enzyme Catalysis
I	Inhibition
L	Molecular Cleavage (includes degradation for Chemicals)
LO	Localization
M	Biochemical Modification
miT	microRNA Targeting
MB	Group/complex Membership
nTRR	Non-Targeting RNA-RNA Interaction
P	Phosphorylation/Dephosphorylation
PD	Protein-DNA binding
PP	Protein-Protein binding
PR	Protein-RNA binding
PY	Processing Yields
RB	Regulation of Binding
RE	Reaction
RR	RNA-RNA Binding
T	Transcription
TR	Translocation
UB	Ubiquitination

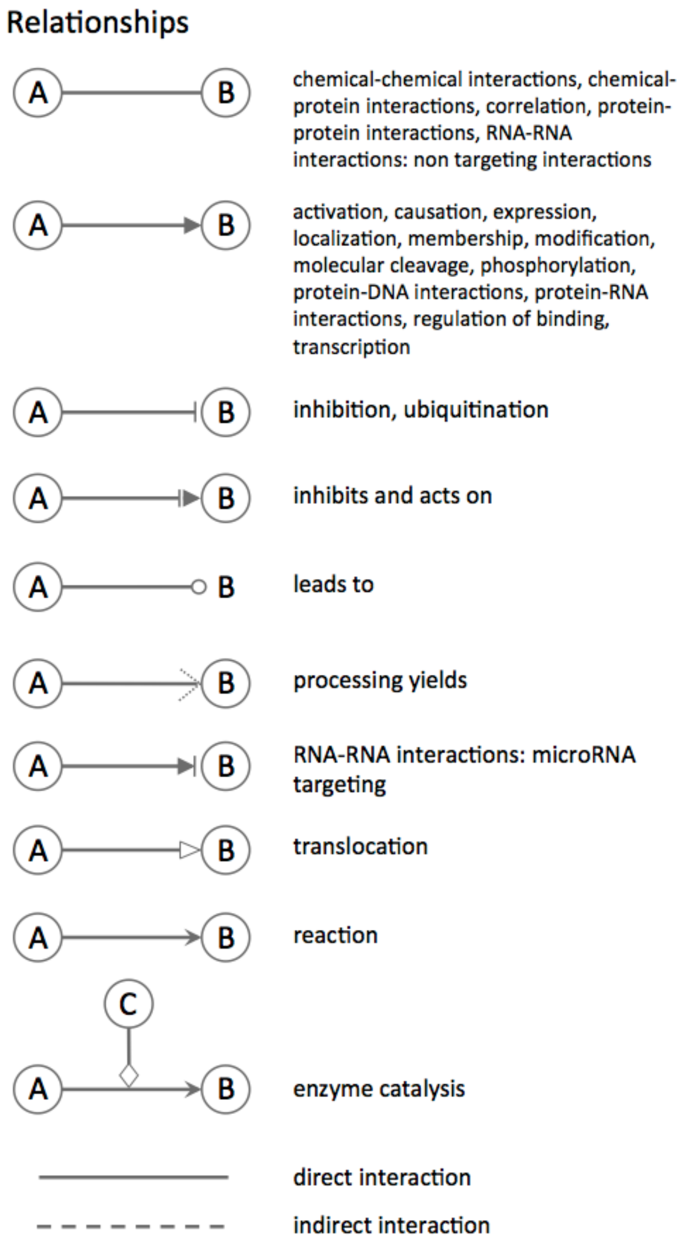

A relationship with an X over it indicates that the interaction does not occur. These relationships are only used in Disease pathways to indicate an interaction that would normally happen in the absence of the disease, but does not happen in the disease context.

An arrow pointing from A to B signifies different actions for different circumstances, as described below:

For signaling pathways:

An arrow pointing from A to B signifies that A causes B to be activated (includes any direct interaction: e.g. binding, phosphorylation, dephosphorylation, etc).

For metabolic pathways:

An arrow pointing from A to B signifies that B is produced from A.

For ligands/receptors:

An arrow pointing from a ligand to a receptor signifies that the ligand binds the receptor and subsequently leads to activation of the receptor. This binding event does not necessarily directly activate the receptor; activation of the receptor could be caused by events secondary to the ligand/receptor binding event.

MAP Prediction Legend

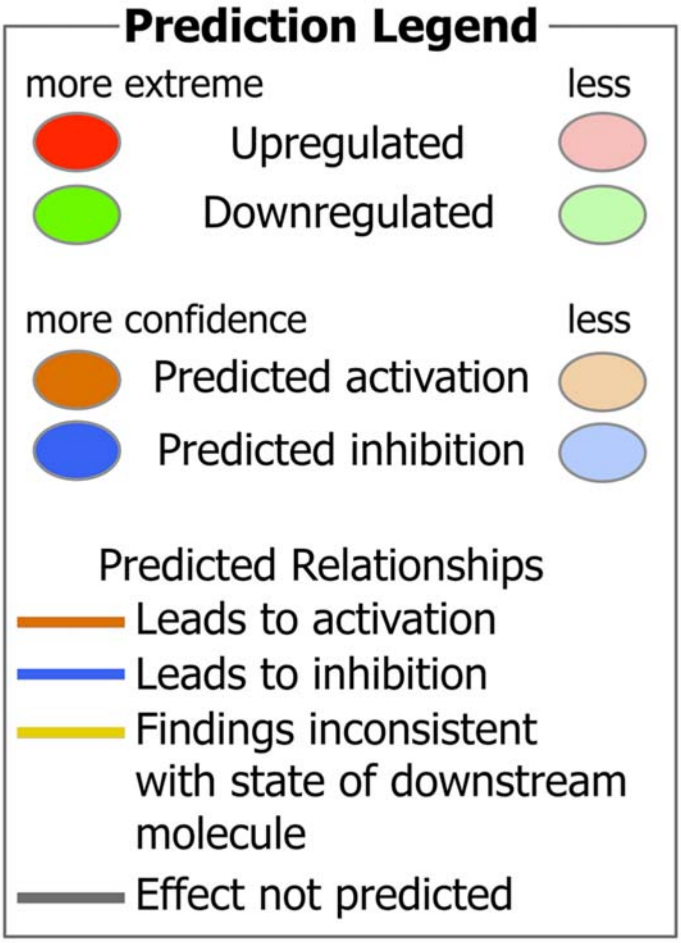

Network and Pathway Overlay Tags

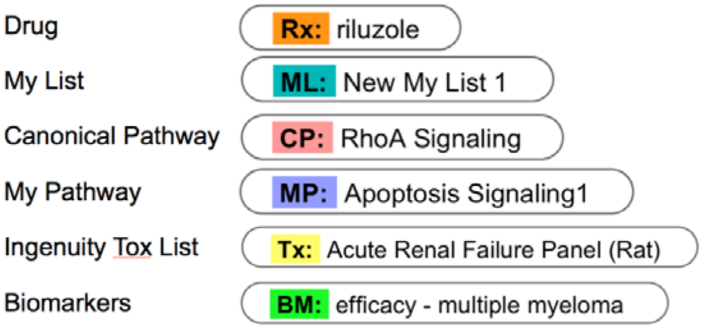

Fonts and Colors

Fonts

**Bold**	Focus molecules. Gene/Protein/ Chemical identifiers that made the user-defined cutoff and map to the Global Molecular Network are displayed with bold text.
Italics	Override molecule - Gene/ Protein/ Chemical identifier designated as Override in the dataset file.
*	Duplicates -Gene/ Protein/ Chemical identifiers marked with an asterisk indicate that multiple identifiers in the dataset file map to a single gene/ chemical in the Global Molecular Network. After an expression value, this indicates that when the duplicates were resolved, a new expression value was calculated that was not part of the original dataset. Please see **Resolving Duplicate Identifiers** for more information.
A	Gene/ Protein/ Chemical ID marked as Absent. The gene/ protein/ chemical will not be used as a focus molecule or appear in networks unless you also explicitly override this flag with the override column.
+	Indicates there are other networks from the analysis that contain this gene. Right click on the + sign or on the corresponding molecule to view the related networks.
Δ	Molecules marked with the Δ (delta) have undergone a change from a previous content release. Changes include: (1) the merging of two or more molecules into one (2) the split of one molecules into two or more molecules (3) the deletion of an obsolete molecule name.
†	The † (dagger) symbol indicates custom molecules.
§	Indicates that the molecule was imported from an SMBL pathway and is not mapped in IPA.

Molecule fill colors on networks and pathways

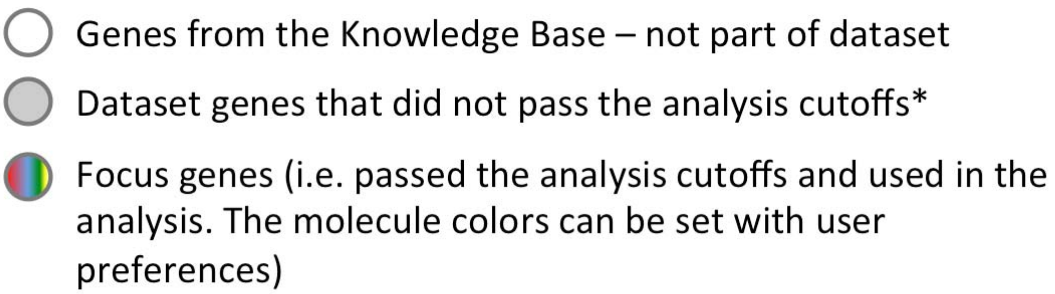

*For datasets that contain only identifiers (i.e. no expression values), the gray fill color identifies the focus genes from that dataset.

Molecule outline colors on networks and pathways

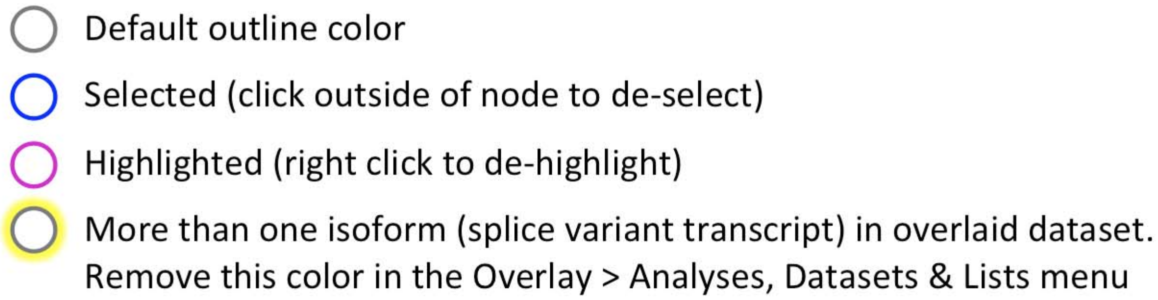

Molecule color intensity: The intensity of green and red molecule colors indicates the degree of down or upregulation, respectively. If normalized ratio, fold-change or log ratio/log fold-change is chosen as Expression Value type, a greater intensity of green represents a higher degree of downregulation, and a greater intensity of red represents a higher degree of upregulation. In contrast, for the expression value type p-value all Focus molecules are red by default; and a higher color intensity represents a lower (and thus more significant) p-value.

Molecule coloring can be customized by adjusting the Application Preferences.

For more information on molecule coloring in Canonical Pathways, click here.

Tool Icons

Button	Name and Function
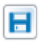	**Save:** Saves the current network diagram.
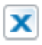	**Delete Selected:** Removes selected (highlighted) nodes from the network diagram.
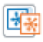	**Copy:** Saves highlighted molecules to the clipboard.
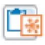	**Paste**: Returns molecules in the clipboard back to a network diagram.
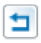	**Undo:** Reverse last action.
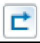	**Redo:** Repeats the last undone action.
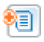	**Add to List**: Selected molecules can be added from a network, pathway, or neighborhood by clicking this icon.
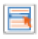	**Graph Options.** *This icon is only available on Mac computers.* It functions as a means to open the right click context menu.
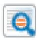	**Find Genes**: Locates a gene within a network, neighborhood, or My Pathway. Once identified, the gene will be identified in dark pink.
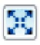	**Full Screen View**: Fill the entire monitor with the current pathway. Hit Escape to return to normal view.
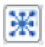	**Layout Options:** Choose from several different layouts, including radial, organic, circular, hierarchical and subcellular.
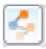	**PathTracer:** Highlight relationships and nodes of interest within networks and pathways by fading away more distant nodes
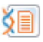	**View annotations:** Gives details of all the selected molecules
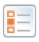	**View/ Edit Preferences:** Click to change the font sizes in network explorer. Changing the font size does not change the corresponding molecule size.
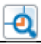	**Zoom Selected:** Zooms in on selected area of window.
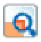	**Magnifying Lens:** Use this loupe tool to enlarge portions of pathways and networks. Click the button then hover with the mouse over the area you wish to see in more detail.
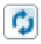	**Refresh:** Updates existing connections with the latest content from the Ingenuity Knowledge Base.
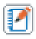	**Edit Notes:** Allows you to change the notes on a saved Pathway or List.
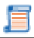	**Open Report:** Opens a Pathway or List report.
BUILD	**Build:** Opens a menu at the left of the screen for accessing Grow, Path Explorer (shortest path), Connect, Trim, and Add Molecules/ Relationships.
OVERLAY	**Overlay**: Opens a menu at the left of the screen for overlaying Expression values, Functions and Diseases, Lists, Drugs, Canonical Pathways, the MAP (Molecule Activity Predictor) and Custom Pathways and for Highlight and Species/ Tissue Highlight.
PATH DESIGNER	**Path Designer:** Use Path Designer to transform your networks and pathways in IPA into publication quality pathway graphics rich with color, customized text and fonts, biological icons, organelles, and custom backdrops.
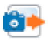	**Export Image:** Export an image of your network up to 600 dpi.
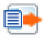	**Export Data**: Export a list of genes with associated identifiers, expression values and gene details.
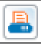	**Print:** Generates a printed copy of the network diagram.
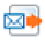	**Email**: Send interactive pathway to a colleague

Graph Navigation Tools

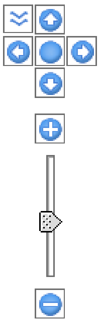

This tool is available from networks and pathways. The arrow keys allow you to move the network left, right, up, and down. The + button acts as a Zoom In tool. The "-" button is a Zoom Out tool. Clicking the circle in the center Fits the network to the screen.

Clicking the the double arrow in the top left opens an overview of the network so that you can determine where on the page you are focusing.

Path Designer Tools

Button	Name and Function
	**Molecules:** Opens the Molecules menu for selecting shape style.
	**Lines:** Draw free lines on your Path Designer pathway.
	**Edges:** Allows you to connect two molecules.
	**Text:** Allows you to type free text on your Path Designer pathway.
	**Cell Art**: Opens the Cell Art menu for selecting organelles and other cellular structures.
	**Legend:** Links to the edit Legend menu that allows you to place a customized legend on your Path Designer pathway.
	**Background:** Links to the edit Background menu that allows you to choose or upload a custom background for your Path Designer pathway.
	**Edit:** Opens a panel on the right of the screen for accessing the details of the Path Designer tools.
	**Text Color**: Changes the color of selected text.
	**Fill:** Allows you to choose and modify the color of the background, text background, or molecule.
	**Line Color:** Allows you to change the color of a free line or relationship.
	**Line Thickness:** Allows you to change the weight of a free line or relationship.
	**Line Style:** Allows you to change the style of a free line or relationship.

Attachment

Help Currency USD
