# Supplementary figures and images for: Olfactory Bulb Proteomics Reveals Widespread Proteostatic Disturbances in Mixed Dementia and Guides for Potential Serum Biomarkers to Discriminate Alzheimer Disease and Mixed Dementia Phenotypes
Source: J Pers Med. 2021 Jun 3;11(6):503. doi: 10.3390/jpm11060503 (PMC8227984; doi:10.3390/jpm11060503)

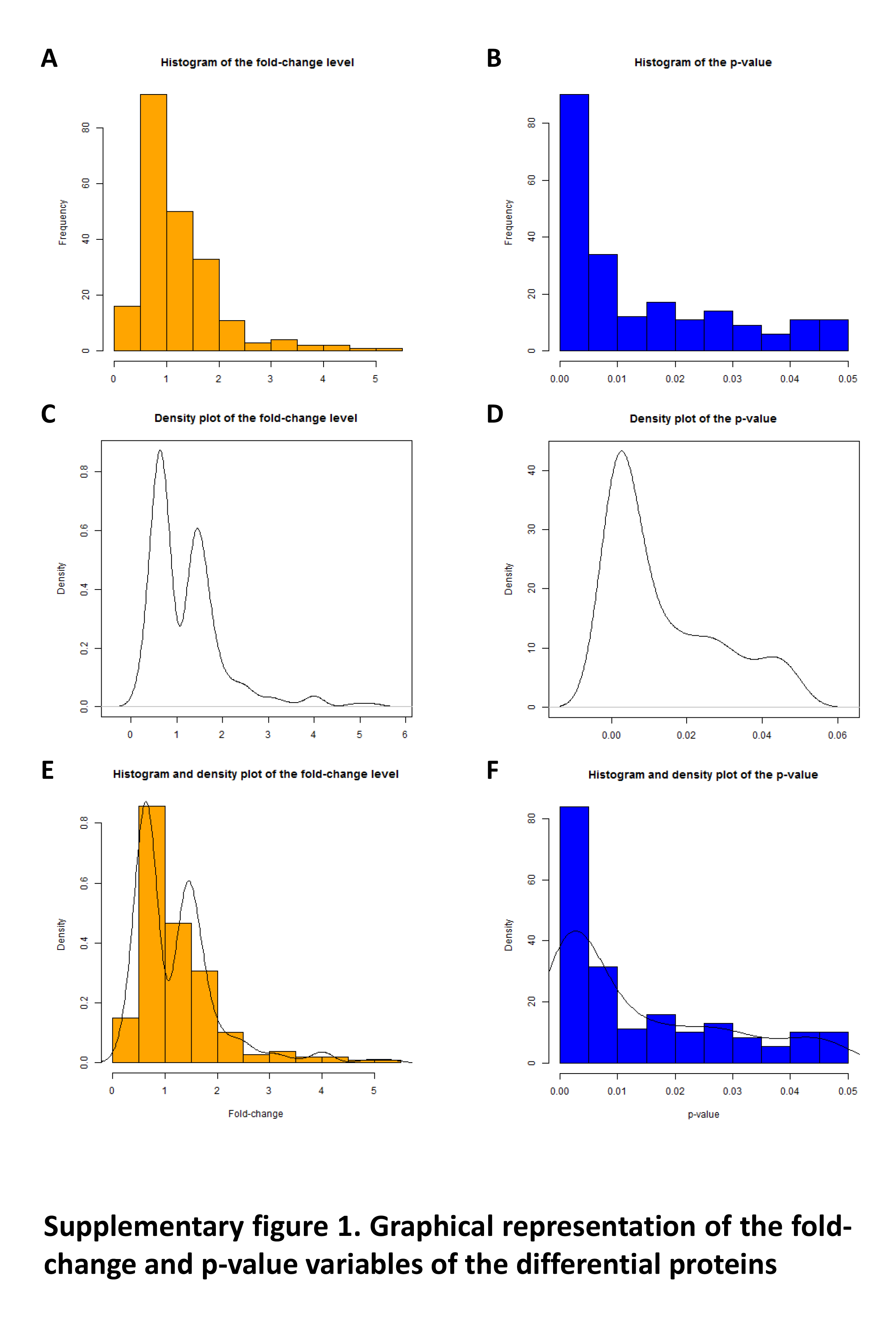

Supplement: Supplementary file 1 [file jpm-11-00503-s001.zip › SUPPLEMENTARY FIGURES AND TABLES/Supplementary Figure 1 20x30cm 300ppp (1).tif]

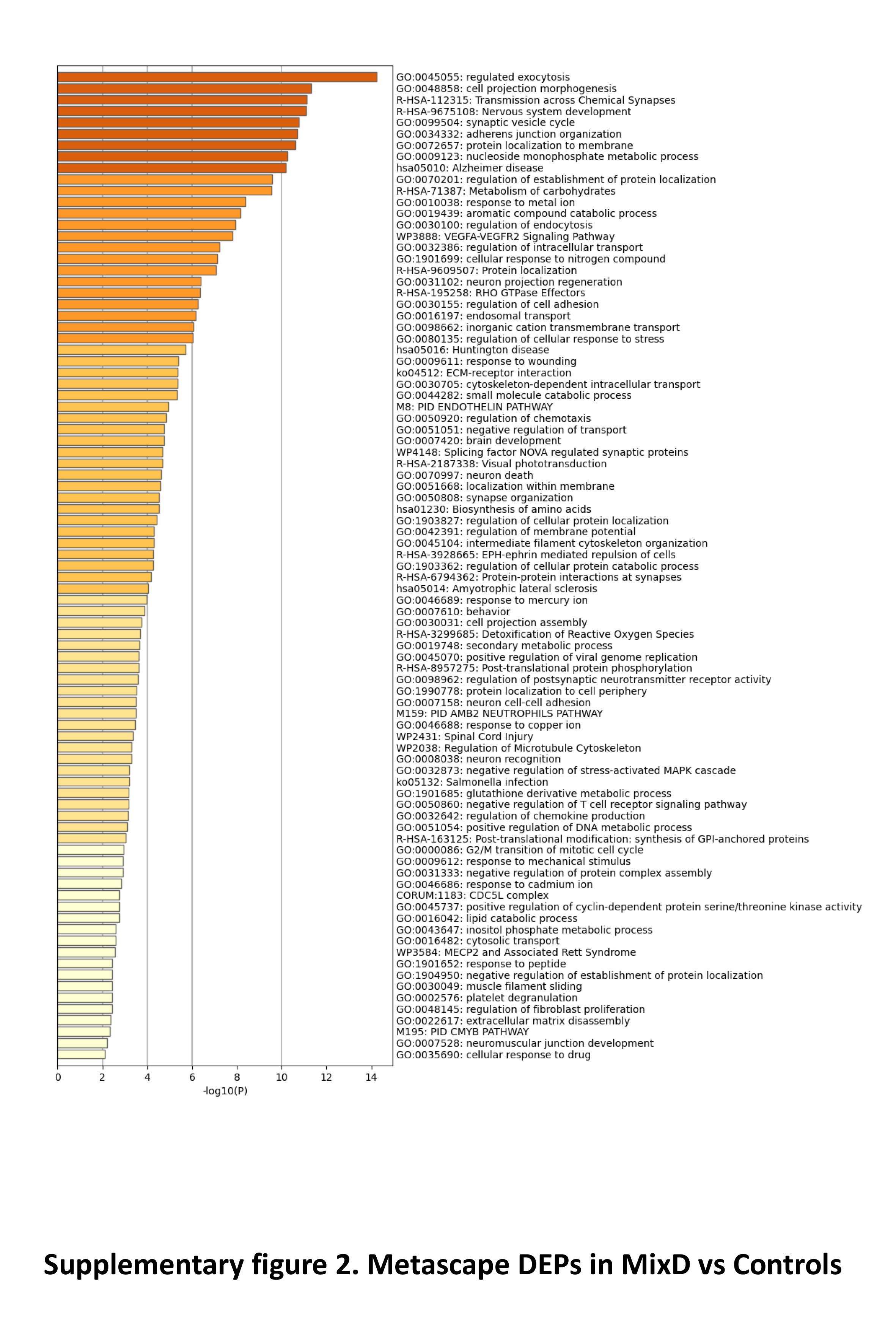

Supplement: Supplementary file 1 [file jpm-11-00503-s001.zip › SUPPLEMENTARY FIGURES AND TABLES/Supplementary Figure 2 20x30cm 300ppp (1).tif]

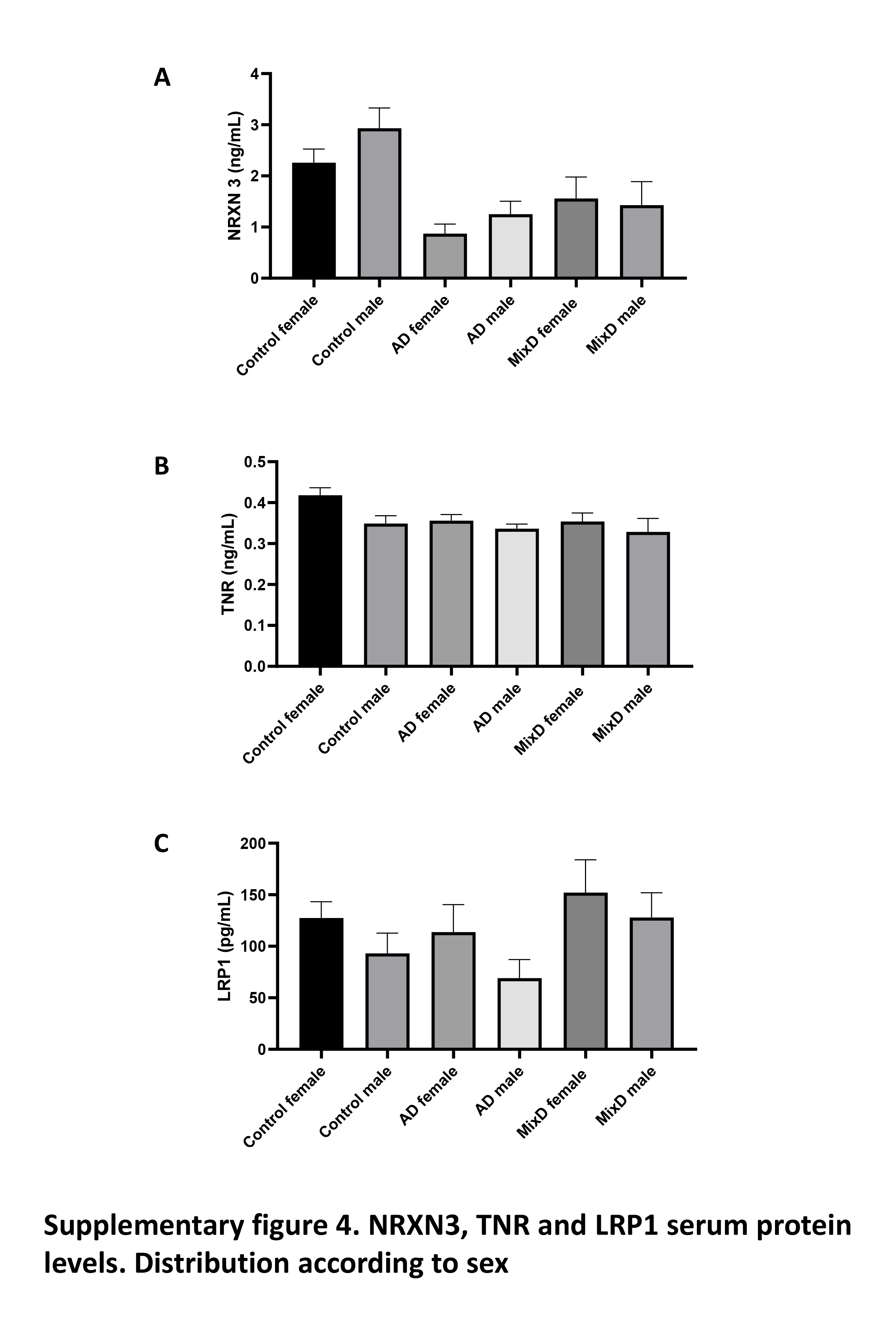

Supplement: Supplementary file 1 [file jpm-11-00503-s001.zip › SUPPLEMENTARY FIGURES AND TABLES/Supplementary Figure 4 20x30cm 300ppp (1).tif]

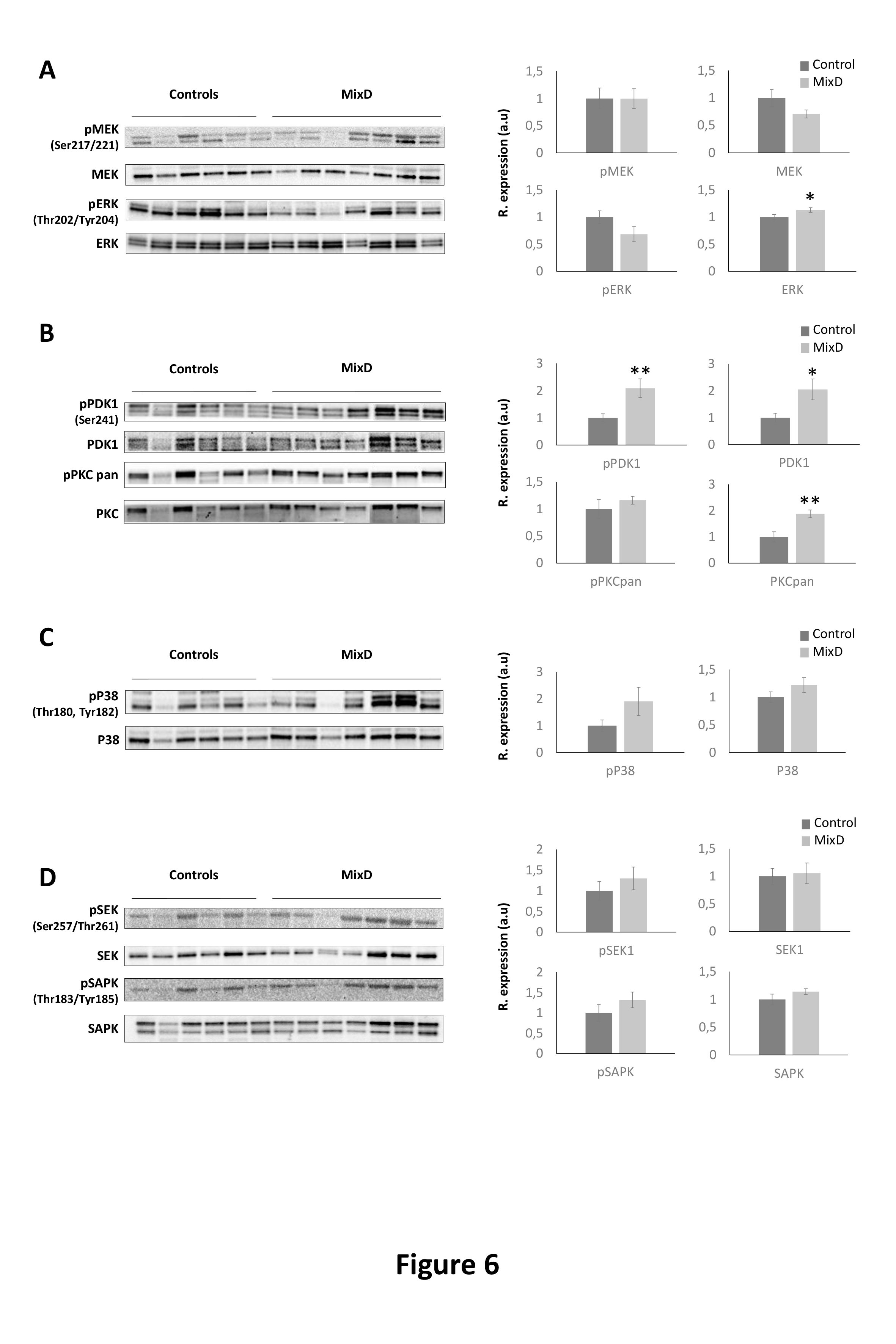

Supplement: Supplementary file 1 [file jpm-11-00503-s001.zip › SUPPLEMENTARY FIGURES AND TABLES/SUPPLEMENTARY FIGURE 3.tif]
